# Assessment of the usefulness of prognostic Van Nuys Prognostic Index in the treatment in ductal carcinoma in situ in 15-year observation

**DOI:** 10.1038/s41598-021-02126-0

**Published:** 2021-11-22

**Authors:** Michał Kunkiel, Anna Niwińska

**Affiliations:** Department of Breast Cancer and Reconstructive Surgery, Maria Sklodowska-Curie National Research Institute of Oncology, W.K. Roentgena 5, 02-781 Warsaw, Poland

**Keywords:** Oncology, Cancer

## Abstract

Ductal carcinoma in situ, a marginal fraction of the mammary gland diseases, is recognized in 25% of breast cancers detected with mammographic screening. The aim of the study was to assess the prognostic value of Van Nuys Prognostic Index, serving to determine the method of treatment according to the recurrence risk. From the 737 of cases of DCIS detection patients treated in our department in the years 1996–2011. The remaining 525 patients whose treatment began from excision of local DCIS and whose further course of treatment was determined after histopathological examination, who were left for observation, treated with lumpectomy and radiotherapy or mastectomy, were qualified for the assessment of mentioned index (Online Appendix 1- Figure S1). The 5-, 10- and 15- year disease recurrence-free survival for the group of 525 patients was 88%, 74% and 62%, respectively. The percentage of 5-, 10- and 15-year disease recurrence-free survival in patients treated in compliance with the VNP Index in individual risk groups did not differ in a statistically significant way. In the low-risk group the percentage of recurrences after local excision, after 5, 10 and 15 years of observation amounted to 8.8%, 22.8% and 28.8%. In patients from this group, the recurrence risk after breast conserving treatment and mastectomy was 2% and 0%, respectively. VNP Index is not an optimal tool for patients with DCIS. It can be helpful only in some clinically difficult cases as one of methods of assessing the risk of recurrence.

## Introduction

Ductal carcinoma in situ (DCIS) used to account for a marginal fraction of the mammary gland diseases. With the commonness of mammographic screening, it has come to constitute approximately 20—25% of breast cancers detected with this method^1–3^. DCIS has thus emerged as an epidemiological problem. Meanwhile difficulties persist in foreseeing its clinical course due to insufficient information on the biology and risk factors of local progression^3^.

The methods of treating DCIS include excision of the lesion without radiation therapy, excision of the lesion with adjuvant radiotherapy (breast conserving therapy) and mastectomy. In part of patients, adjuvant hormone therapy is applied. Research carried out into the treatment of DCIS patients in numerous countries worldwide revealed a great variety of therapeutic procedures as well as a change of trends in treatment over time^4,5^.

The histoclinical prognostic factors distinguished include: patient age, DCIS size, grade of nuclear malignancy, presence of *comedo,* histological type, multifocality, size of healthy tissue margin around the excised lesion, the way of DCIS detection (clinically explicit vs mammography alone detectible)^6^. The prognostic indices developed so far attempted to combine the most essential histoclinical risk factors and thus enable the choice of the optimal therapy. The most outstanding among them are Nomogram Memorial Sloan – Kettering Cancer Centre (MSKCC)^7^, The University of Southern California Van Nuys Prognostic Index (USC/VNPI)^8–11^, Oncotype DX DCIS^12,13^ and the NCCN prognostic index^14^, each of them having their limitations and none of them having been declared the optimal one.

The University of Southern California/Van Nuys Prognostic Index^9^, published in 2003 constitutes a numerical algorithm which allows to assess the recurrence risk and indicates the optimal method of treatment. The independent, clinically essential recurrence risk factors analysed in this nomogram include: tumour size, patient age, width of the surgical margin and grade of nuclear malignancy as well as the presence or absence of *comedo-*type necrosis^9^. Each of the features is assigned a score from 1 to 3 where score 1 is the most clinically favourable value while score 3 constitutes an unfavourable prognostic factor. The sum of all the scores gives a final result of 4 to 12. A score ranging from 4—6 signifies a low recurrence risk group and constitutes an indication for the performance of a local excision alone, a score of 7—9 is an indication for adjuvant radiotherapy after excision (breast conserving treatment) while a score of 10 -12 suggests the necessity of performing mastectomy^9^. The VNPI was developed on the basis of a repeatedly up-dated and modified retrospective analysis of patients treated for DCIS, patients not being subjected to randomization as regards the method of treatment^8–11^.

**Figure 1:**
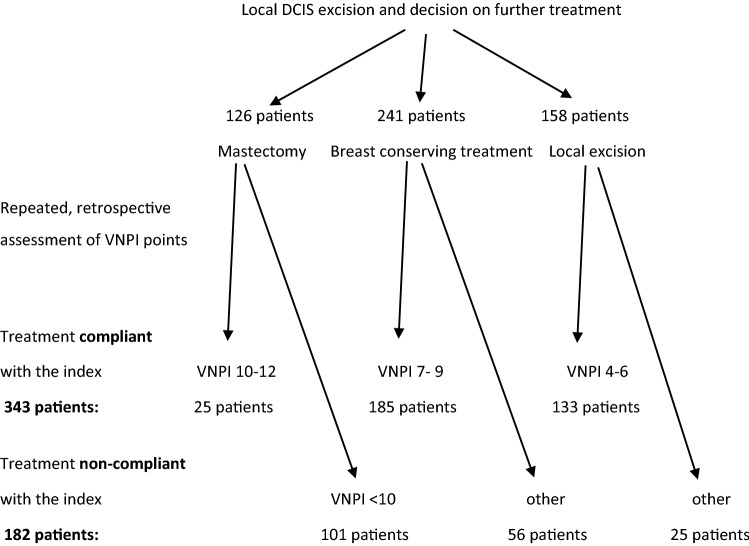
Material of 525 patients according to repeated, retrospective assessment and division into VNPI risk groups.

The scheme (principles) of the 2003 index are presented in Fig. 1. The application of the VNPI in individual countries varies. In Australia and New Zealand, 77% of DCIS patients are treated in compliance with the VNPI recommendations^15^. On the other hand, in Great Britain this percentage is merely 15.8%^16^.

In the years 1996–2011, the National Institute of Oncology—Public Research Institute [NIO-PRI] in Warsaw, Poland, used subsequent versions of the VNPI in the treatment of DCIS patients.

The aim of the study was to assess the prognostic value of The University of Southern California/Van Nuys Prognostic Index (UC/VNPI), published in 2003, serving to determine the method of treatment according to the recurrence risk^9^.

## Methods

The study was retrospective in character and all methods were carried out in accordance with relevant guidelines and regulations (permission was obtained from the Bioethical Committee of the NIO-PRI in Warsaw, also informed consent for the study participation was obtained from the patients). From the 737 of consecutive cases of DCIS patients treated in the National Institute of Oncology—Public Research Institute in Warsaw in the years 1996–2011, 211 patients who underwent primary mastectomy without conservative treatment were excluded from the study. Those were patients with an extensive, more than 4 cm DCIS on mammography, with multifocal calcifications on mammography, after double non-radical excision of the lesion or after primary mastectomy performed at a patient's special request irrespective of the DCIS size.

The remaining 525 consecutive patients whose treatment began from excision of a local DCIS and whose further course of treatment was then determined after a histopathological examination, who were left for observation, treated with lumpectomy and radiotherapy or mastectomy, were qualified for the assessment of the VNPI according to the 2003 criteria^9^. Since in the period of 16 years, from 1996 to 2011, the method of treating patients in the clinic underwent modifications in accordance with consecutive guidelines of the VNPI authors as well as individual decisions of the medical team, the assessment of the VNIP score in the 525 patients after breast conserving therapy was repeated retrospectively to assess the value of the VNPI of 2003 analysing 4 risk factors. What was obtained were groups of patients with the same score treated in or without compliance with the VNPI recommendations. The differences allowed to compare the methods of treatment of patients with the same VNPI score and to see whether the choice of the method of treatment for individual risk groups proposed by the authors of the 2003 VNPI is optimal^9^. Tables 1, 2 gives the characteristics of the group of 525 patients.

**Table 1:** Clinical characteristics of the group of 525 patients.

Features	Number of patients (percentage %)
Number of patients	525 (100)
Patient age (range, mean)	23–85, 57
< 50	121 (23)
50–57	152 (29)
58–64	126 (24)
> 64	126 (24)
**DCIS diagnosed**	
On mammography alone	472 (89.9)
Clinically and mammographically	53 (10.1)
**Cancer size on mammography (cm)**	
< 0.5	98 (18.7)
0.5–1	139 (26.5)
01-Feb	152 (29.0)
> 2	136 (25.9)
**Differentiation grade G**	
NG1	132 (25.2)
NG2	195 (37.3)
NG3	196 (37.5)
No data	2
***Comedo* type necrosis**	
Present	333 (63.7)
Absent	190 (36.3)
No data	2
**Healthy tissue margin around the excised DCIS (mm)**	
< 0.2	142 (27.0)
0.2–0.6	129 (24.6)
0.6–1	128 (24.4)
> 1.5	126 (24.0)
**Type of procedure**	
Mastectomy	126 (24)
Breast conserving treatment	241 (45.9)
Local excision	158 (30.1)
**Treatment in accordance with the VNPI [45]**	
Yes	343 (65.3)
No	182 (34.7)

**Table 2:** VNPI modified in 2003.

Risk factor	Score 1	Score 2	Score 3
Tumour size (mm)	≤ 15	16–40	≥ 41
Margin width (mm)	≥ 10	01-Sep	< 1
Histopathological classification	NG1/NG2 with *comedo* necrosis	NG1/NG2 with *comedo* necrosis	NG3 with or without *comedo* necrosis
Age (years)	≥ 60	40–60	< 40
Total of scores	Method of treatment		
Score 4—6	Local excision		
Score 7—9	Local excision + radiation therapy		
Score 10—12	Mastectomy		

Radiotherapy after conservative breast treatment was performed in 241 patients. Over the 16 years covered by the study, radiation from oblique fields with gamma rays Co 60 of 1.25 MV or 4 MV or 6 MV photons generated from a linear accelerator was applied to the patients' whole breast. A fraction dose of 2 Gy to a total dose of 50 Gy (32 patients) or a fraction dose of 2.25 Gy to a total dose of 45 Gy or a fraction dose of 2.5 Gy to a total dose of 42.5 Gy was applied. An increase in the dose on the bed of the excised lesion, of 5 fractions of 2 Gy to a total of 10 Gy, was applied in 58 (24%) of the patients with unfavourable prognostic factors.

None of the patients in the total group was treated with an adjuvant hormone therapy with tamoxifen.

All the patients completed the treatment 6–21 years ago. The median for the duration of observation for the whole group with a 95% confidence interval (114–126 months) was 120 months.

The VNPI score was repeatedly, retrospectively, calculated in a group of 525 patients. Next, patients were selected in accordance with the VNPI score^9^: patients with score 4, 5 and 6 who underwent an excision of the lesion alone (low-risk recurrence group), patients with score 7, 8 or 9 after breast conserving treatment (middle-risk recurrence group) and patients with score 10, 11 or 12 after mastectomy (high-risk recurrence group). The remaining patients, treated with other-than-recommended methods, were also divided into risk groups with scores 4–6, 7–9 and 10–12 (patients treated without compliance with the VNPI recommendations). The DFS and the cumulated percentage of recurrences in individual groups (with scores 4–6 vs 7–9 vs 10–12) treated in compliance or without compliance with the VNPI recommendations were compared. It was checked which of the methods of treatment in individual risk groups was more advantageous for patients: that following the VNPI recommendations or that chosen by a doctor on the basis of individual risk assessment. The second stage consisted in an analysis of the role of radiotherapy in the low-risk recurrence patient with 4–6 VNPI points. The percentage of recurrences after excision without radiotherapy (treated in compliance with the VNPI index) and after excision with radiation therapy (group treated without compliance with the VNPI 2003 protocol) was compared. The last stage involved an analysis on the basis of the Cox proportional hazard model to assess the prognostic value of individual recurrence risk factors comprised in the VNPI index in the group of patients treated in compliance with the VNPI index.

Standard descriptive statistics tools were used to describe the material. The disease-free time (DFS) and the overall survival (OS) were calculated with the use of the Kaplan–Meier method and the cumulated percentage of recurrences with the competing risks method with death for other reasons as the competing risk. In order to assess the usefulness of the VNPI in its 2003 version, the results of the local excision. breast conserving treatment and mastectomy in the three VNPI groups with the index values of 4–6, -9 and 10–12 were compared. In addition, for the group of treated in compliance with the VNPI recommendations, an analysis of prognostic factors for the risk of failure was carried out with the use of the Cox proportional hazard model. In the process of modelling, stepwise variables-elimination method with standard inclusion (0.05) and exclusion (0.1) thresholds was used. The set of analysed factors included: patient age, method of DCIS diagnosis (mammography alone vs clinically explicit), DCIS size, nuclear malignancy grade (NG), presence of type *comedo* necrosis and width of healthy tissue margin around the excised DCIS. All the estimates were given with 95% confidence intervals. Critical test values below 0.05 were adopted as statistically significant. The IBM SPSS Statistics 23.0.0.2 statistical packet and R version 3.4.4. were employed in the analysis.

## Results

Figure 1 presents the group of 525 DCIS patients after the repeated calculation of the number of VNPI scores. 343 patients treated in compliance with the VNPI (133 patients with score 4–6 after local excision, 185 patients with score 7–9 after breast conserving breast treatment and 25 patients with score 10–12 after mastectomy) were distinguished. The remaining 182 patients were not treated in compliance with the VNPI recommendations.

The 5-, 10- and 15- year disease recurrence-free survival (DFS) for the group of 525 patients was 88%, 74% and 62%, respectively. The percentage of 5-, 10- and 15-year DFS in patients treated in compliance with the VNPI in individual risk groups did not differ in a statistically significant way, *p* = 0.104.

**Figure 2:**
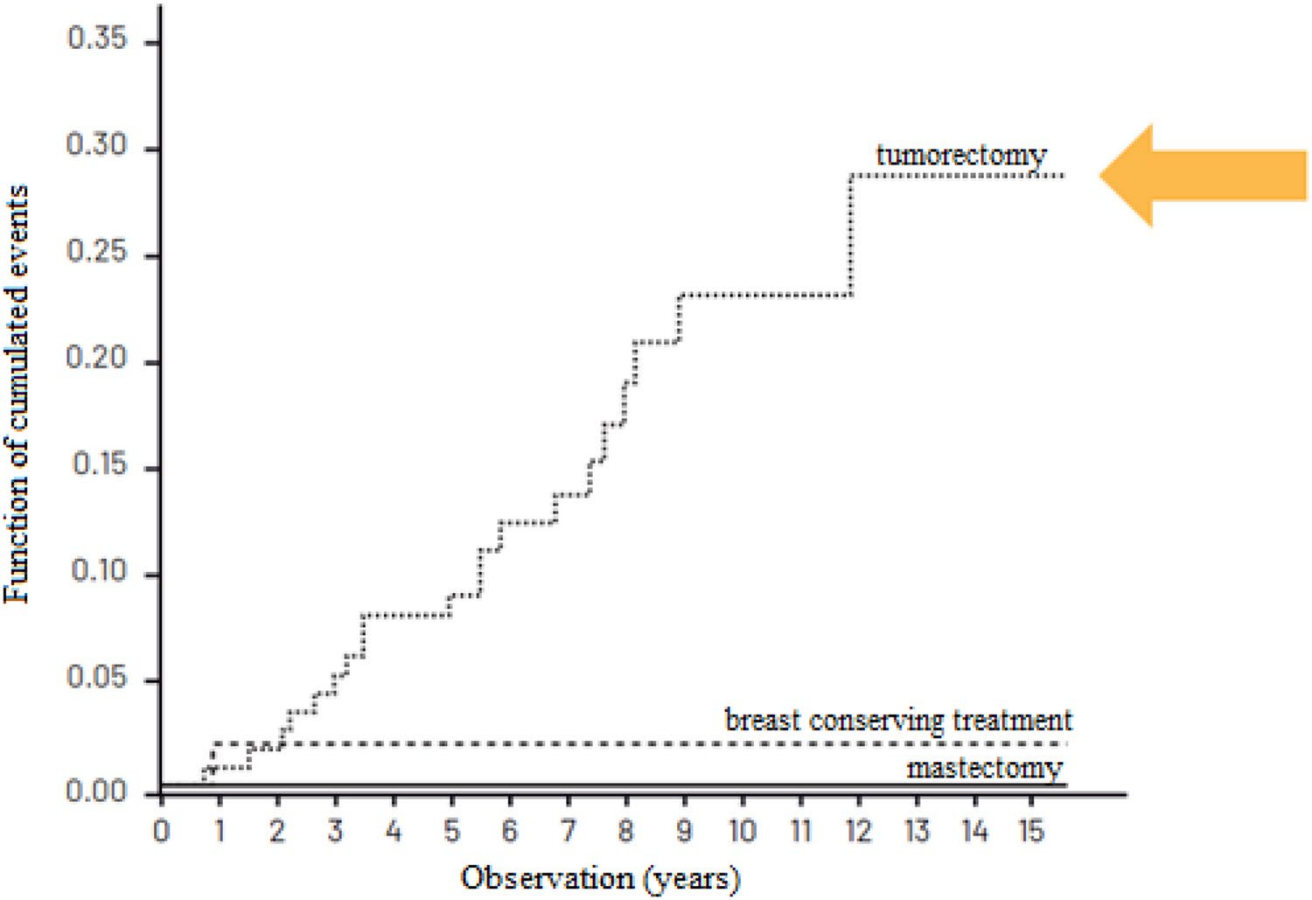
Cumulated recurrence risk in the low-risk group (4–6 VNPI points) with division into the method of treatment. The arrow indicates treatment in compliance with the VNPI (BCS).

**Figure 3:**
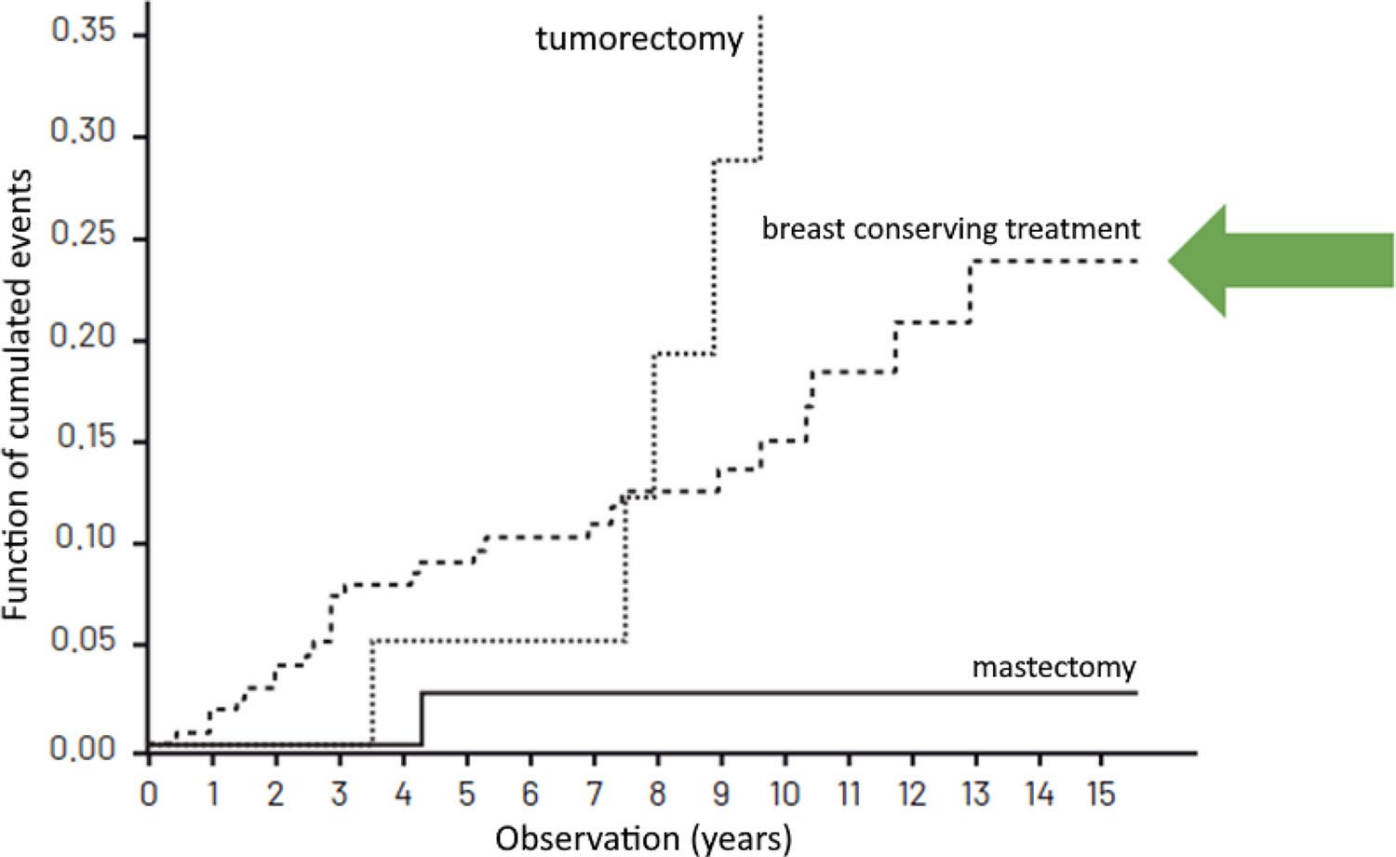
Cumulated recurrence risk in the middle-risk group (score 7–9 VNPI) with division into the methods of treatment. The arrow indicates treatment compliant with the VNPI (BCT).

**Figure 4:**
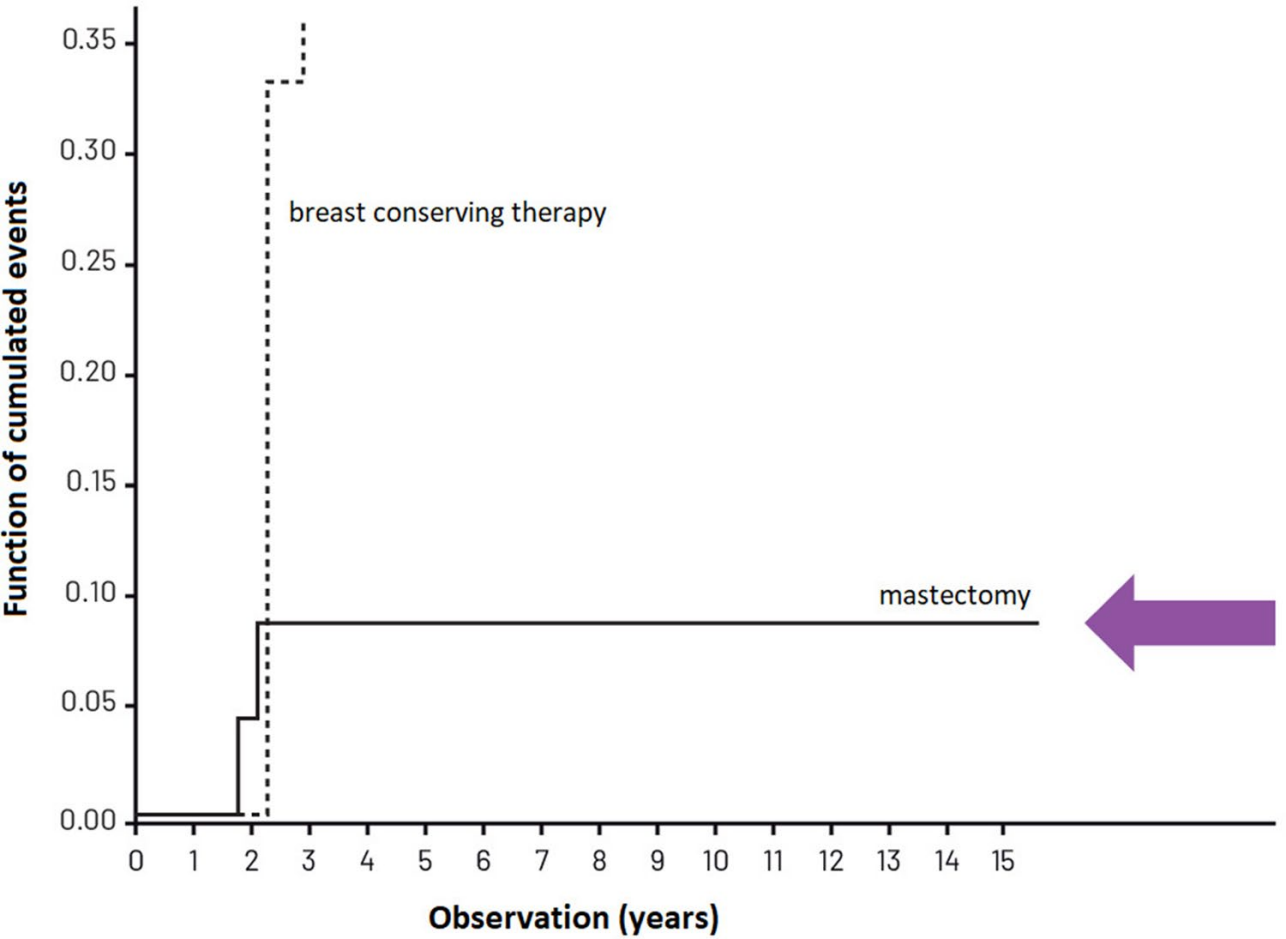
Cumulated recurrence risk in the high-risk group (10—12 VNPI points) with division into the methods of treatment. The arrow indicates treatment compliant with the VNPI (mastectomy).

Table 3 presents the cumulated recurrence risk in patients treated in compliance with the VNPI, after 5, 10 and 15 years of observation while Figs. 2, 3 and 4—the cumulated recurrence risk in the 3 risk groups according to VNPI: Fig. 2—the low-risk group, Fig. 3—the middle-risk group, Fig. 4—the high recurrence risk group. Each of the Figures gives the results of different methods of treatment (compliant and non-compliant with the VNPI for a given risk group. The arrow indicates the method of treatment compliant with the VNPI recommendations for a given risk group.

**Table 3:** Cumulated recurrence risk after 5, 10 and 15 years of observation in patients treated in compliance with the VNPI, *p* < 0.001.

Duration of observation	Mastectomy 10–12 points percentage, 95% CI	Breast conserving treatment 7–9 points percentage, 95% CI	Local excision 4–6 points percentage, 95% CI
5 years	8.7% [0; 20.411]	8.9% [4.734; 13.103]	8.8% [3.576; 14.141]
10 years	8.7% [0; 20.411]	14.9% [8.941; 20.969]	22.8% [12.938; 32.703]
15 years	8.7% [0; 20.411]	23.8% [13.616; 34.176]	28.8% [14.033; 42.910]

In the low-risk group, with score 4, 5 or 6 according to the VNPI, the percentage of recurrences after local excision (treatment compliant with the VNPI recommendations), after 5, 10 and 15 years of observation amounted to 8.8%, 22.8% and 28.8%. In patients from this group, the recurrence risk after breast conserving treatment and mastectomy (treatment non-compliant with the VNPI recommendations) was 2% and 0%, respectively, *p* = 0.012.

In the middle-risk group, with 7, 8 or 9 points, according to the VNPI, the percentage of recurrence after breast conserving treatment (treatment compliant with the VNPI recommendations), after 5, 10 and 15 years of observation amounted to 8.9%, 14.9% and 23.8%. In patients from this group, the recurrence risk after local excision and mastectomy (treatment non-compliant with the VNPI recommendations) was 53% and 2.5%, respectively, *p* = 0.001.

In the high-risk group, with 10, 11 or 12 points, according to the VNPI, the percentage of recurrence after mastectomy (treatment compliant with the VNPI recommendations), after 5, 10 and 15 years of observation amounted to 8.7% which indicated mastectomy as the only method acceptable in this prognostic group. In patients from this group, the recurrence risk after breast conserving treatment (treatment non-compliant with the VNPI recommendations) was 66.7%, respectively, *p* = 0.014.

**Figure 5:**
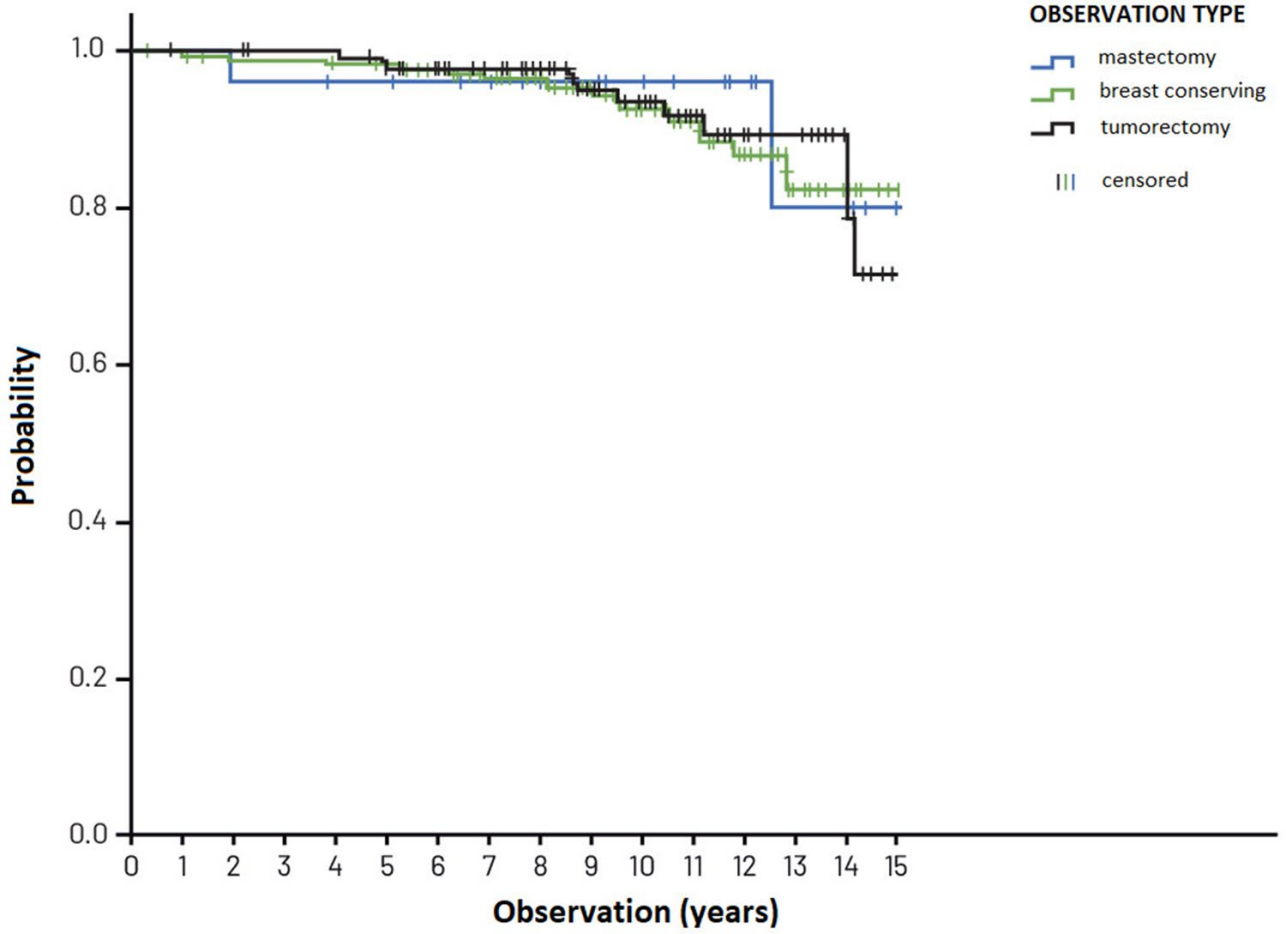
Overall survival curve of patients after BCS (tumorectomy), BCT and mastectomy treated in compliance with VNPI, *p* = 0.872.

The 5-, 10- and 15-year overall survival (OS) in the group of 525 patients amounted to 97%, 92% and 78%, respectively. The overall 15-year survival of patients treated in compliance with the VNPI, in the three risk groups, that is after mastectomy, breast conserving treatment and local excision was 78%, 82% and 72%, respectively, and did not differ in a statistically significant way, *p* = 0.872. Figure 5 presents overall survival curves for patients in the risk groups, treated in compliance with the VNPI recommendations.

### Assessment of the value of radiation therapy in patients after local DCIS excision in the low-risk group.

**Figure 6:**
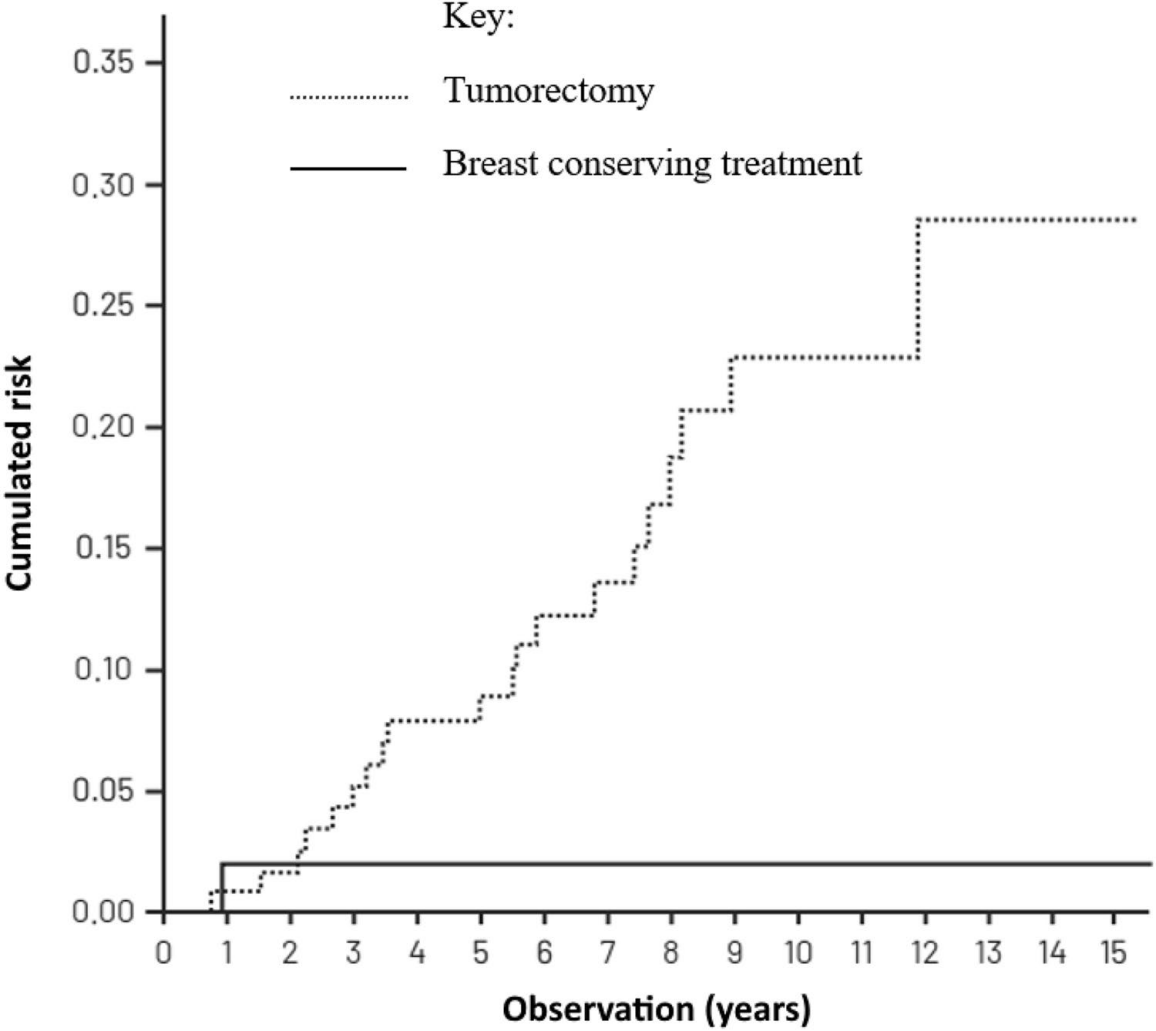
Cumulated recurrence risk in patients with score 4, 5 or 6 treated in compliance with (BCS) and without compliance with (BCT) the VNPI recommendations, *p* < 0.001.

In the group of 158 patients with a low recurrence risk (score 4, 5 and 6 points according to the VNPI), DFS, the cumulated recurrence risk and the OS were compared in patients after local excision alone (treatment in compliance of VNPI recommendations, 133 patients) as well as after the addition of radiotherapy (non-compliant treatment, 25 patients). DFS in patients after DCIS excision, after 5-, 10- and 15-years of observation amounted to 87%, 65% and 51%, respectively, and after breast conserving treatment- 98%, 90% and 86%, respectively (*p* = 0.002). Figure 6 present the cumulated recurrence risk in patients in the low risk group after local excision and after breast conserving treatment. The recurrence risk was significantly higher in patients without radiotherapy. After 15 years of observation, the difference amounted to 26.8% (2% after radiotherapy vs 28.8% without radiotherapy). The pooled data with confidence intervals are presented in online appendices (Tables S1, S2 and S3).

**Figure 7:**
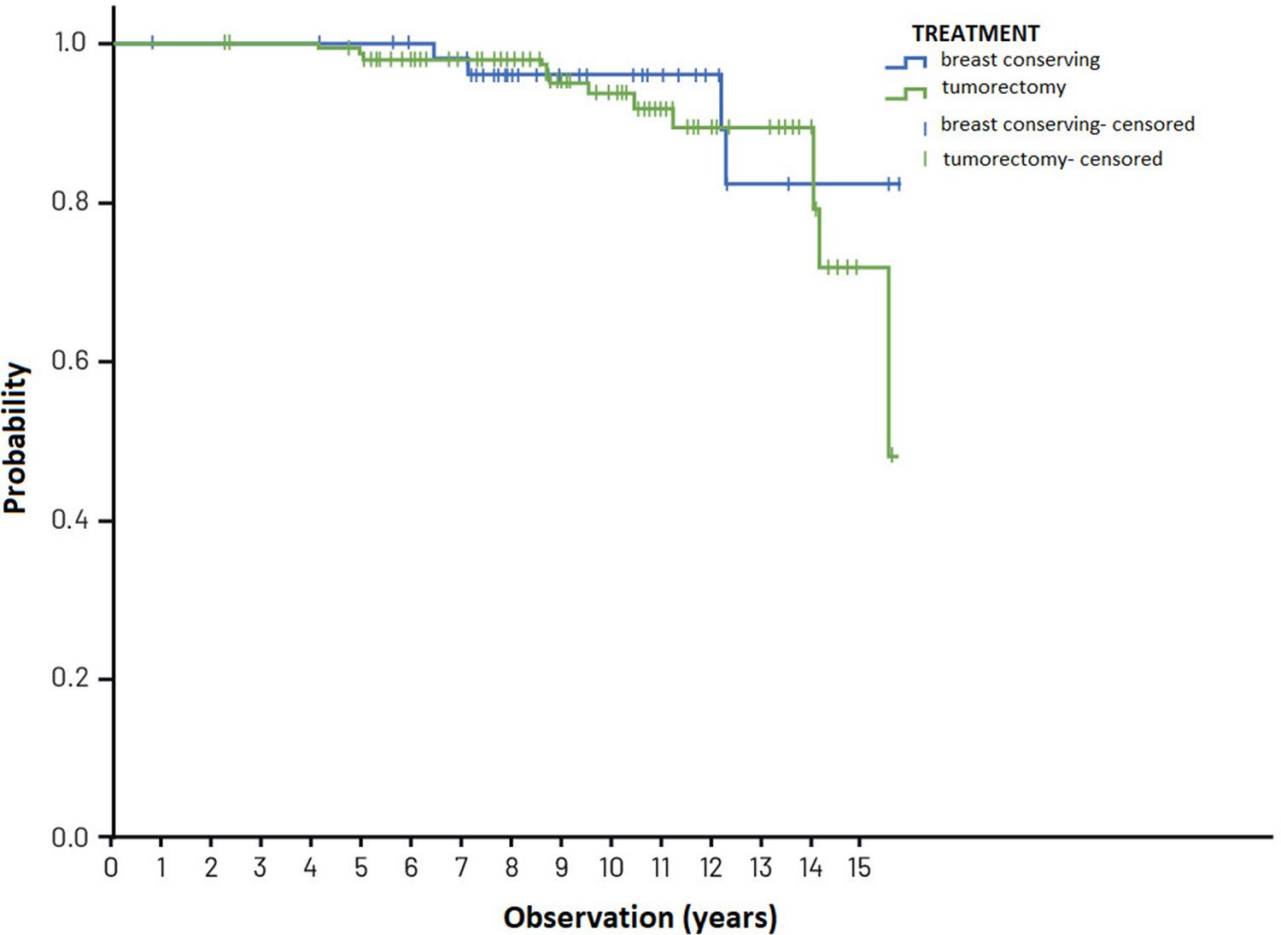
Overall survival (OS) in the low risk group with 4–6 points, after local excision and breast conserving treatment, *p* = 0.403.

The 5-, 10- and 15- year OS in the low-risk group treated in compliance with the VNPI was 98%, 94% and 72%, respectively, and in the group treated without compliance with the VNPI—100%, 96% and 82%. The difference in OS was not statistically significant (*p* = 403). Figure 7 presents the overall survival in patients with 4–6 points treated with and without compliance with the VNPI.

### Assessment of the prognostic value of the VNPI prognostic factors

The Cox analysis of the risk-of-failure function did not reveal a statistically significant influence solely for the *comedo* type necrosis, with the presence of necrosis linked to a better prognosis (B = -0.51, SD = 0.240, *p* = 0.033, HR = 0.601, 95CI 0.376; 0.960). The remaining 5 factors were not found to be of statistically significant influence on the risk of failure. The p value for the remaining 5 factors was p > 0.1.

## Discussion

In the years 1996–1997, DCIS patients in the NIO-PRI in Warsaw, Poland, were treated in different ways. However, after 2003, the prevailing majority of patients were treated in compliance with the VNPI of 2003^9^. In order to establish the practical importance of the VNPI of 2003, groups of patients treated in compliance with and without compliance with the VNPI were distinguished and the percentage of recurrences was compared. It was compared which method of treatment applied in individual groups was more advantageous for the patients: that in compliance with the VNPI recommendations or that chosen by a doctor on the basis of an individual recurrence risk assessment.

In the group of 133 low recurrence-risk patients, with score 4, 5 or 6, treated in the NIO-PRI in compliance with the VNPI (local excision) a surprisingly unfavourable result was reported—28.8% of recurrences in 15-year observation. The result differed significantly from the percentage of recurrences described by Silverstein (7.5% after 12 years)^11^. Simultaneously, radiotherapy in 56 patients with score 4–6, which was not compliant with the VNPI, and applied mainly in young women with NG3 or *comedo* type necrosis, decreased that risk to 2% in the 15 years of observation. High effectiveness and validity of the application of radiotherapy in this group of patients was thus proved.

In the middle-recurrence risk group, with score 7, 8 or 9, the method proposed by Silverstein was breast conserving treatment^9^. In Silverstein's analyses, the recurrence risk after DCIS excision alone in this group amounted to 27% while after radiotherapy it was 17% lower. In the presented study, the percentage of recurrences after breast conserving treatment (treatment in compliance with the VNPI) in the middle risk group after 15 years of observation amounted to 23.8% while after excision alone it was significantly higher than in the study of Silverstein and amounted to 52.6%. The validity of the application of radiotherapy after a breast conserving surgery in the middle-risk group was proved, but the still high percentage of recurrences is evidence of the presence in this group of a part of patients who should undergo mastectomy.

In the high recurrence risk group, with score 10, 11 or 12, the validity of the performance of mastectomy (treatment in compliance with the VNPI) was confirmed as breast conserving treatment resulted in a very high (66.7%), non-acceptable, percentage of recurrences while mastectomy reduced the recurrence risk in 15-year observation to 8.7%.

In the presented study, the high percentage of local recurrences did not affect the overall survival rate.

The assessment of the clinical effectiveness of the VNPI index was performed by few retrospective studies^17–20^ and the results of those analyses are negative or not unambiguous. Gilleard and others^17^ analysed the clinical value of the VNPI on a group of 215 patients after a breast conserving treatment alone. The Index was based on an analysis of 3 risk factors (the 1996 VNPI version). The results of the analysis suggested that the VNPI is a useful tool for the stratification of patients in terms of the recurrence risk. It allowed to distinguish a low-risk group in which no recurrences were observed during 8 years of observation. Boland and others^18^ made an analysis of the VNPI on a group of 237 British DCIS patients treated with breast conserving method, with a 47-month long median observation. The results of the study did confirm a sufficient ability of the VNPI to distinguish risk groups. The study conducted by MacAusland and others^19^ analysed the recurrence risk after 5 years of observation in 222 patients after DCIS excision, depending on the number of VNPI points published in the years 1996 and 2003 as well as exclusively on the size of the healthy tissue margin (3 analyses). On the division into three risk groups according to the VNPI of 2003, the recurrence risk in the low, middle and high-risk group was 5%, 17% and 0%, respectively. The study failed to confirm the value of the VNPI in the assessment of the recurrence risk and the selection of the right treatment of patients. Di Saverio and others analysed retrospectively the results of the treatment of 259 DCIS patients dividing patients into risk groups according to the VNPI of 2003^20^. The results and conclusions were not unambiguous. The authors did not find statistically significant differences between the risk groups. In 2015, the author of the VNPI himself up-dated his results on the basis of an analysis of 1 704 DCIS patients^11^. In 385 patients with score 4, 5 or 6, the percentage of recurrences after local excision alone after 12 years of observation amounted to 7.5% while after excision and radiotherapy to 3.6% (*p* = 0.33). The authors maintained their position as regards absence of indications for radiotherapy in this group. Patients with score 10, 11 or 12 had an at least 40% lower recurrence risk and consequently qualified for mastectomy. After mastectomy, that risk was 7%. Simultaneously, changes had place in the middle-risk group with score 7—9 points. Patients with margins of < 3—5 mm had a high recurrence risk and consequently, in spite of the initial VNPI classification for breast conserving treatment, according Silverstein, were to be sent for mastectomy^11^. That update of the recommendations was evidence of the fact that the VNPI proposed in 2003, based on 4 risk factors, was not optimal.

Numerous authors pointed out that a prospective study with randomization should be performed to determine the repeatability and usefulness of the VNPI^17–22^, but the only RTOG study begun, intending to answer the question, was closed due to lack of recruitment^23^.

Contemporary knowledge about the recurrence risk in DCIS patients casts doubt on the ranges of each of the four risk factors set in 2003^9^ which were assigned a specific score (Table 2). This concerns in particular the assignment of 2 points. The same score is given to women of 41 and 59, patients with DCIS size of 1.6 and 4 cm as well as patients with a margin of 1 mm and 9 mm in spite of the fact that the patients differ considerably in prognostic terms^6,23^. In light of randomized studies, a DCIS of < 2.5 cm is considered prognostically promising while a DCIS of > 2.5 cm as an adverse risk factor^21^. Similarly, a healthy tissue margin around an excised DCIS of < 2 mm is not acceptable unless the patient is undergoing a breast conserving treatment and the size of the margin around the DCIS after excision without radiotherapy is not known at all^24^. The above, more recent, data found in literature raise doubts about the system of calculation of the VNPI score used so far.

Radiotherapy significantly decreased the recurrence risk in DCIS patients after local excision. Radiotherapy in the low-risk group (score 4, 5 and 6) which does not require this treatment according to the VNPI, resulted in a reduction of the recurrence risk of 26.8% in 15-year observation (28.8% of recurrences without radiotherapy and 2% after radiotherapy), however without an impact on the overall survival. The result confirms the advantage of radiotherapy even in the group with the best prognosis. This denies the assumptions of the VNPI and confirms the results of 5 clinical studies with randomization which document the advantages of radiotherapy in all DCIS patients after a breast conserving treatment. The studies proved that radiotherapy improves the results of treatment in all DCIS patients after a breast conserving treatment. Its advantageous impact is proportional to the grade of the recurrence risk and increases with the observation time (results also presented by McCormick et al. during 2018 ASTRO Annual Meeting Late-breaking Abstract Selections LBA1 Randomized Trial Evaluating Radiation following Surgical Excision for “Good Risk” DCIS: 12-Year Report from NRG/RTOG 9804)^25–30^. The high percentage of recurrences in the low risk group, reported in this study is evidence of the inability of the VNPI to successfully select patients who could safely avoid radiation therapy.

## Conclusions

The VNPI published in 2003 is not an optimal tool for the choice of DCIS patients^9^. It can be helpful only in some clinically difficult cases as one of the many methods of assessing the risk of DCIS recurrence. The ranges concerning age, DCIS size and margin size which were given a specific numerical value are not consistent with the current research findings and recommendations of scientific societies. The attempt at determining the low risk group in which could be safely resigned from radiotherapy after a breast conserving treatment with the help of the VNPI has failed. An overwhelming majority of DCIS patients after a breast conserving surgery should receive radiotherapy and the decision to resign from radiation therapy should be made by an interdisciplinary team, together with the patient. However, despite the high risk of recurrence in the low-risk group according to the VNPI, we did not see an impact on OS. The current challenge for oncologists, surgeons and radiotherapists is to search for risk factors for recurrence, not in the form of DCIS, but as an invasive recurrence.

## Supplementary Information

Supplementary Figure 1.

Supplementary Table 1.

Supplementary Table 2.

Supplementary Table 3.

Supplementary Table 4.

Supplementary Table 5.
