# Supplementary material for: Assessment of the usefulness of prognostic Van Nuys Prognostic Index in the treatment in ductal carcinoma in situ in 15-year observation
Source: Sci Rep. 2021 Nov 22;11:22645. doi: 10.1038/s41598-021-02126-0 (PMC8608918; doi:10.1038/s41598-021-02126-0)
Supplement: Supplementary file 1 — Supplementary Figure 1. [file 41598_2021_2126_MOESM1_ESM.docx]

**525 patients**

**BCT with DCIS and the decision on further treatment**

126 patients 241 patients 159 patients

Mastectomy BCT Lumpectomy

Treatment **according to Index** VNPI 10-12 VNPI 7-9 VNPI 4-6

345 patients: 25 patients 185 patients 133 patients

Treatment **not** **according to Index** VNPI <10 other other

182 patients: 101 patients 56 patients 25 patients

Figure S1. The decision on the further treatment in patients with DCIS who underwent BCT.
