# Supplementary material for: Assessment of the usefulness of prognostic Van Nuys Prognostic Index in the treatment in ductal carcinoma in situ in 15-year observation
Source: Sci Rep. 2021 Nov 22;11:22645. doi: 10.1038/s41598-021-02126-0 (PMC8608918; doi:10.1038/s41598-021-02126-0)
Supplement: Supplementary file 2 — Supplementary Table 1. [file 41598_2021_2126_MOESM2_ESM.docx]

| **Type of treatment** | **5 yrs % DFS**  **95%- confidence intervals** | **10 yrs % DFS**  **95%- confidence intervals** | **15 yrs % DFS**  **95%- confidence intervals** |
| --- | --- | --- | --- |
| **VNPI 4, 5, 6** | | | |
| Mastectomy | **86.1**  76.1 - 96.1 | **77.1**  64.2 – 90.0 | **56.6**  42.6 – 70.6 |
| BCT | **98.0**  94.0 - 100 | **85.9**  73.5 – 98.3 | **85.9**  73.5 – 98.3 |
| Lumpectomy | **86.9**  80.9 – 92.9 | **64.7**  52.7 – 76.7 | **50.5**  31.5 – 69.5 |
| **VNPI 7, 8, 9** | | | |
| Mastectomy | **92.6**  84.4 –100 | **92.6**  84.4 –100 | **92.6**  84.4 –100 |
| BCT | **88.3**  83.3 – 93.2 | **74.4**  62.2 – 82.4 | **59.2**  47.2 – 71.1 |
| Lumpectomy | **71.3**  69.3 – 73.1 | **47.5**  21.6 – 68.4 | **23.8**  0 – 60.0 |
| **VNPI 10, 11, 12** | | | |
| Mastectomy | **91.3**  79.5 – 100 | **91.3**  79.5 – 100 | **76.1**  47 - 100 |
| BCT | **33.3**  0 – 87.7 | **33.3**  0 – 87.7 | **33.3**  0 – 87.7 |

Table S1. 5, 10 and 15-year relapse-free survival, DFS in 525 patients by group risks and treatment. The results of treatment of patients treated according to the index are marked in color VNPI.
