# Supplementary material for: Assessment of the usefulness of prognostic Van Nuys Prognostic Index in the treatment in ductal carcinoma in situ in 15-year observation
Source: Sci Rep. 2021 Nov 22;11:22645. doi: 10.1038/s41598-021-02126-0 (PMC8608918; doi:10.1038/s41598-021-02126-0)
Supplement: Supplementary file 3 — Supplementary Table 2. [file 41598_2021_2126_MOESM3_ESM.docx]

| **VNPI/method of the treatment** | **8 years- PFS** | **95%- confidence interval** | **12 years- PFS** | **95%- confidence interval** | **p** |
| --- | --- | --- | --- | --- | --- |
| **VNPI 4, 5 or 6**   - mastectomy - BCT - lumpectomy | 0,812  0,904  0,753 | 0,696-0,926  0,812-0,996  0,640-0,849 | 0,716  0,859  0,856 | 0,552 - 0,880  0,735 - 0,983  0,736 - 0,976 | **0,012** |
| **VNPI 7, 8 or 9**   - mastectomy - BCT - lumpectomy | 0,926  9,824  0,570 | 0,844-1  0,7440,890  0,330-0,810 | 0,926  0,648  0,238 | 0,844 - 1  0,542 - 0,754   1. - 0,6 | **<0,001** |
| **VNPI 10, 11 or 12**   - mastectomy - BCT | 0,913  0,333 | 0,795-1  0-0,877 | 0,913  0,333 | 0,795 - 1  0 - 0,877 | **0,014** |

Table S2. Probability of asymptomatic living for 8 and 12 years.
