# Supplementary material for: Assessment of the usefulness of prognostic Van Nuys Prognostic Index in the treatment in ductal carcinoma in situ in 15-year observation
Source: Sci Rep. 2021 Nov 22;11:22645. doi: 10.1038/s41598-021-02126-0 (PMC8608918; doi:10.1038/s41598-021-02126-0)
Supplement: Supplementary file 4 — Supplementary Table 3. [file 41598_2021_2126_MOESM4_ESM.docx]

| **VNPI/method of the treatment** | **8 years- CIF** | **95%- confidence interval** | **12 years- CIF** | **95%- confidence interval** | **p** |
| --- | --- | --- | --- | --- | --- |
| **VNPI 4, 5 or 6**   - mastectomy - BCT - lumpectomy | 0  0,020  0,189 | -  0-0,058  0,103-0,276 | 0  0,020  0,288 | -  0 - 0,058  0,143 - 0,432 | **0,012** |
| **VNPI 7, 8 or 9**   - mastectomy - BCT - lumpectomy | 0,250  0,125  0,193 | 0-0,074  0,074-0,176  0-0,402 | 0,025  0,208  0,526 | 0- 0,074  0,122 - 0,294  0,027 - 1,025 | **<0,001** |
| **VNPI 10, 11 or 12**   - mastectomy - BCT | 0,087  - | 0-0,204  - | 0,087  - | 0 - 0,204  - | **0,014** |

Table S3. Accumulated recurrence function for 8 and 12 years
