# Supplementary figures and images for: Assessment of the usefulness of prognostic Van Nuys Prognostic Index in the treatment in ductal carcinoma in situ in 15-year observation
Source: Sci Rep. 2021 Nov 22;11:22645. doi: 10.1038/s41598-021-02126-0 (PMC8608918; doi:10.1038/s41598-021-02126-0)

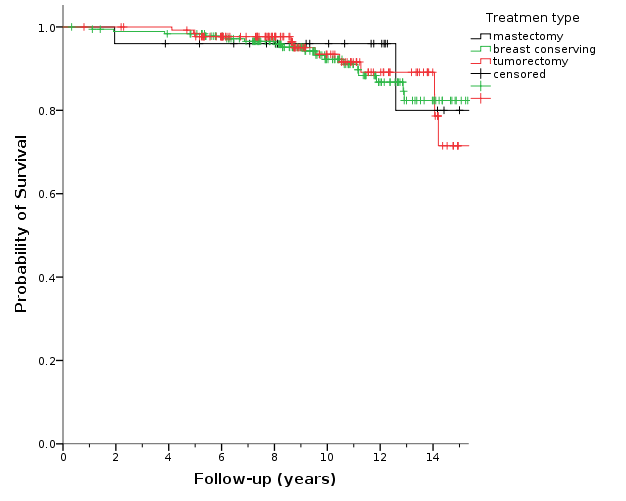


| Mastectomy | 25 | 24 | 23 | 22 | 18 | 13 | 9 | 4 |
| --- | --- | --- | --- | --- | --- | --- | --- | --- |
| BCT | 185 | 179 | 177 | 162 | 137 | 86 | 50 | 28 |
| Tumorectomy | 133 | 131 | 129 | 113 | 86 | 53 | 31 | 16 |

Figure S2. Survival VNPI compliance.

Supplement: Supplementary file 5 — Supplementary Table 4. [file 41598_2021_2126_MOESM5_ESM.docx]

| BCT | 52 | 52 | 52 | 48 | 34 | 22 | 14 | 8 |
| --- | --- | --- | --- | --- | --- | --- | --- | --- |
| Tumorectomy | 133 | 131 | 129 | 113 | 86 | 53 | 31 | 16 |

Figure S3. Survival low risk treatment.


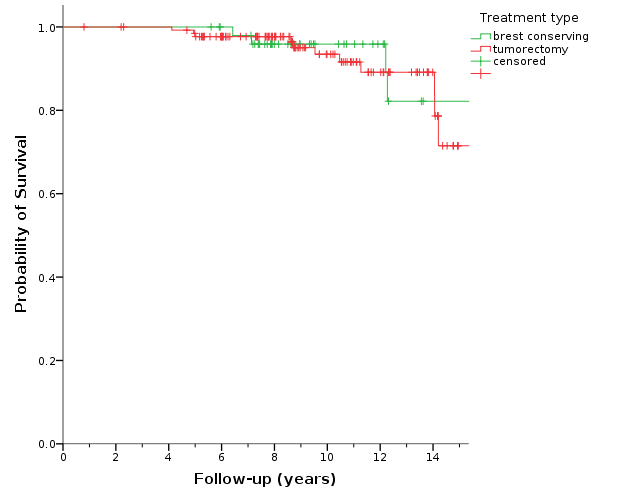

Supplement: Supplementary file 6 — Supplementary Table 5. [file 41598_2021_2126_MOESM6_ESM.docx]
